# Descemet's Stripping Automated Endothelial Keratoplasty versus Descemet's Membrane Endothelial Keratoplasty in the Fellow Eye for Fuchs Endothelial Dystrophy: A Retrospective Study

**DOI:** 10.1155/2015/750567

**Published:** 2015-11-09

**Authors:** Vipul Bhandari, Jagdeesh K. Reddy, Kirti Relekar, Vijayalakshmi Prabhu

**Affiliations:** Sankara Eye Centre, Coimbatore 641035, India

## Abstract

*Aim*. To evaluate visual outcome and endothelial cell density (ECD) after Descemet's Membrane Endothelial Keratoplasty (DMEK) in comparison with Descemet's Stripping Automated Endothelial Keratoplasty (DSAEK) in the fellow eye for Fuchs endothelial dystrophy (FED).* Design*. Single-centre, retrospective case series.* Methods*. 30 eyes of 30 patients undergoing DMEK, who completed a 1-year follow-up, were compared with 30 fellow eyes which underwent DSAEK for bilateral FED. Main outcome measures studied included Best Corrected Visual Acuity (BCVA) and ECD during a 1-year follow-up period.* Results*. BCVA improved from 0.78 ± 0.35 logMAR, and 0.73 ± 0.31 logMAR before surgery to 0.22 ± 0.1 logMAR and 0.35 ± 0.12 logMAR 6 months after DMEK and DSEK, respectively (*P* < 0.001). At one year after surgery, the BCVA was maintained at 0.21 ± 0.12 logMAR and 0.34 ± 0.1 logMAR, respectively, after DMEK and DSAEK. A statistically better visual outcome was observed after DMEK compared to DSAEK (*P* < 0.05) in fellow eyes.* Conclusions*. DMEK provided better visual rehabilitation when compared to DSAEK. Nevertheless, there were no significant differences with regard to the ECD within a 1-year follow-up.

## 1. Introduction

The last decade has witnessed a revolutionary shift in the treatment of corneal endothelial disease [1]. Commencing with the advent of posterior lamellar keratoplasty in the late 1990s, a number of procedures have been developed, refined, and widely adopted, which have given patients faster recoveries and improved globe stability in comparison to traditional corneal transplantation. Each iteration of endothelial keratoplasty (EK) has involved the increasingly selective transplantation of corneal endothelial cells [2]. This was possible because most patients who need corneal surgery suffer from diseases that are restricted to only one particular layer of the cornea. Many patients undergoing keratoplasty suffer from disorders of the corneal endothelium such as Fuchs endothelial dystrophy (FED) or pseudophakic/aphakic bullous keratopathy [3]. A hospital-based study in South India reported that corneal dystrophies including FED accounted for 8.1% (144 patients) of all keratoplasties performed [4]. In another study involving 2022 penetrating keratoplasties (PK) performed in a tertiary eye care centre in North India, it was found that bullous keratopathy accounted for 13.5% of all operated cases [5]. The first attempt to perform “posterior lamellar keratoplasty” (PLK) was described in 1950 by Dr. Jose Barraquer [6], who performed PLK after creating a corneal flap. In the modern history of PLK, Melles [7, 8] described sutureless PLK in 1998, where an air bubble is used for fixation of the posterior lamella. In 2001, Terry [9, 10] coined the term “Deep Lamellar Endothelial Keratoplasty” (DLEK). A further improvement of EK was described in 2005 by Price [11, 12], who performed “Descemet Stripping Endothelial Keratoplasty” (DSEK), followed 1 year later by Gorovoy [13], who used a microkeratome and termed this procedure “Descemet Stripping Automated Endothelial Keratoplasty” (DSAEK). In the abovementioned procedure, the diseased endothelium and Descemet's membrane (DM) of a host is replaced with posterior corneal stroma, DM, and endothelium of a donor. Recently, “Descemet's Membrane Endothelial Keratoplasty” (DMEK) was described by Melles et al. [14], which involves transplantation of an isolated endothelium-Descemet's membrane (EDM) layer without adherent corneal stroma. Therefore, DMEK is the only technique that tries to completely retain the anatomy of the recipient's cornea. The main outcome measures that define the success of a surgical procedure for corneal replacement are the functional outcome as well as endothelial survival. Among the outcome measures that can be used to define the success of a surgical procedure like postoperative visual outcome and postoperative astigmatism, several studies have shown that DSAEK and DMEK offer significant advantages over PK and also provide faster functional rehabilitation [8, 15–17]. PK might be superior to DSAEK or DMEK, with respect to endothelial cell survival in the early postoperative period [18, 19]. Mid-term results, however, show comparable endothelial cell survival between PK and DSAEK or DMEK [20–24]. A study evaluating outcomes of DMEK in the fellow eye of patients with DSAEK in the first eye has not yet been carried out in the Indian population. This study aims to evaluate the visual outcomes and endothelial cell density (ECD) in patients undergoing DSAEK in one eye in comparison to DMEK in fellow eye for bilateral FED at a tertiary eye centre in South India.

## 2. Materials and Methods

The study commenced after obtaining the Institutional Ethics and Scientific Committee approval. Thirty eyes of thirty patients undergoing DMEK who completed a 1-year follow-up, were included in this study. Patients with other ocular comorbidity besides FED were not included. All eyes were pseudophakic with a posterior chamber intraocular lens implanted previously. These eyes were compared with 30 fellow eyes of the same patients who underwent DSAEK earlier. Indication for surgery was bilateral FED. All patients received a graft prepared from a corneoscleral button that had been stored in Cornisol (Aurolab, Madurai, India) at 4°C (short-term cultured graft) in coordination with our eye bank. All donor corneas with ECD more than 1800 cells/mm^2^ were used in this study. The corneas were taken from donors aged between 40 and 65 years. Published data by Laaser and associates [25] suggests that donor tissue culture conditions have no significant effect on visual outcome and endothelial cell survival. All surgeries were performed by two surgeons under regional/peribulbar anaesthesia. Donor preparation was performed immediately prior to transplantation.

### 2.1. Surgical Technique of DMEK

**Figure 1:**
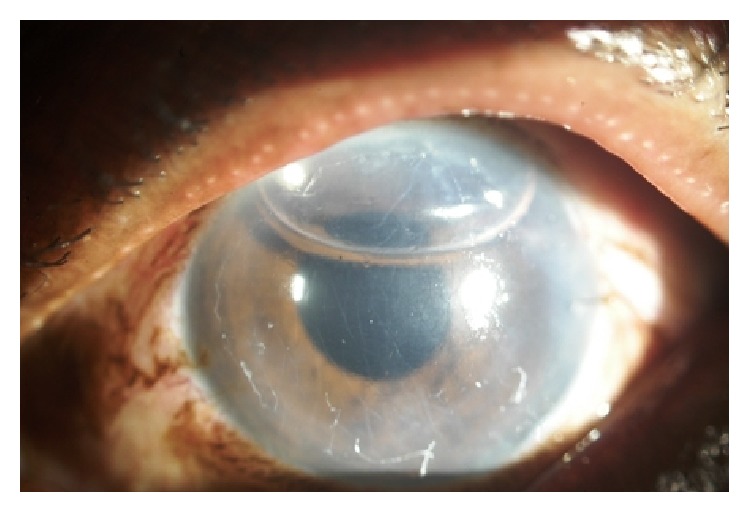
After DMEK.

**Figure 2:**
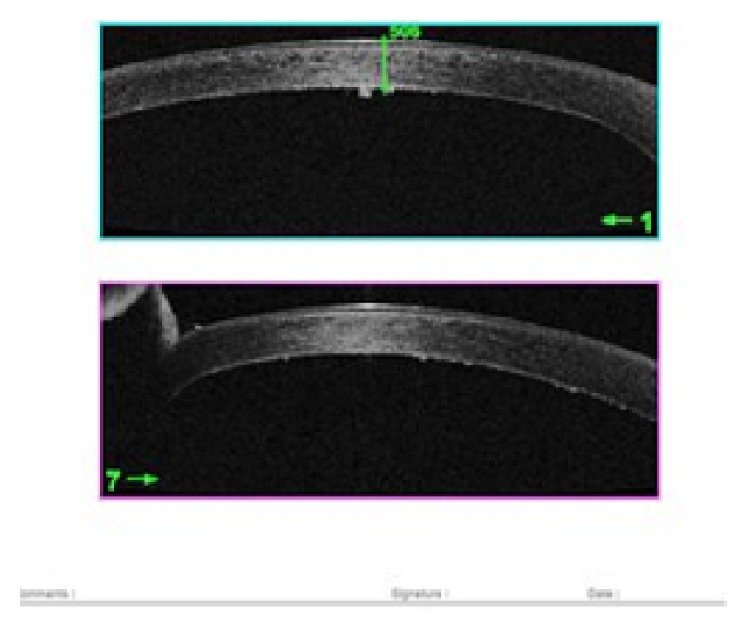
AS-OCT picture after DMEK.

The EDM was stripped from the donor corneal stroma by a technique previously described by Kruse et al. [26], which included several modifications from the original technique described by Melles [14, 27]. After mounting the corneoscleral buttons on a teflon block, the endothelium was marked by gentle touch with 8.0 mm trephine and stained with 0.06% trypan blue (Rhex ID; Appasamy Ocular Devices Pvt Ltd) for 60 seconds. The trephine mark was scored with Sinskey's hook and then the edge was grasped with two forceps. By simultaneous centripetal movement of the 2 forceps, the EDM was completely detached [28]. After marking the epithelium with 8 mm marker, the patient's EDM was removed under air using an inverted hook (reverse Sinskey's hook) inside the 8 mm marking. Graft was injected into the patient's eye by a customised injector made of a silicon hub, Alcon (USA) “C” cartridge with a 1 mL syringe attached to it and unfolding was achieved by a standardized technique due to the elastic properties of DM. The EDM was positioned centrally using small bursts of balanced salt solution (BSS) and unfolded by repeatedly tapping on the surface of the cornea with endothelium down. Air was injected underneath the graft until the anterior chamber (AC) was completely filled with air, which was left in place for 2 hours following the procedure. After 2 hours, air was released in operating room (OR) ensuring that at least half of the AC was still air filled (Figure 1). Anterior Segment Optical Coherence Tomography (AS-OCT) [RTVue Model-RT100 Version 6.9] was used to confirm the DM attachment and its configuration (Figure 2).

### 2.2. Surgical Technique of DSAEK

The preparation of donor corneal lenticules was performed using the ML7 (Med-Logics, California) microkeratome with a 400 *μ* head. The stromal side of the corneal lenticule was marked with 3 spots of different sizes to ensure correct orientation in the anterior chamber of the host. After marking the epithelium with 8 mm marker, the patient's EDM was removed under air using an inverted hook (reverse Sinskey's hook) inside the 8 mm marking. The graft was delivered to the patient's eye using the standardized pull-through technique by Busin et al. [29]. The graft was placed on the plate and pulled into the funnel shaped part of the Busin glide using a micro-incision forceps. The Busin glide was then inverted and positioned at the nasal clear cornea tunnel. On the temporal side, a microincision forceps was inserted to pull the graft into the anterior chamber, allowing it to unfold spontaneously. Air was injected underneath the graft until the AC was completely filled with air, which was left in place for 2 hours following the procedure. After 2 hours, air was released in the OR ensuring that at least half of the AC was still air filled. Main outcome measures included Best Corrected Visual Acuity (BCVA) and ECD during 1-year follow-up. ECD was analyzed using specular microscope (Tomey EM-3000, Tomey Corporation, Japan). The mean central corneal graft thickness and also the central corneal thickness were assessed 6 months postoperatively with AS-OCT.

All patients were started postoperatively on a tapering dose of prednisolone acetate 1% eye drops (Pred Forte, Allergan, Irvine, CA, USA) over a period of 2 months and Vigamox eye drops (preservative free moxifloxacin eye drops, Alcon Lab Inc., Fort Worth, TX, USA) 4 times a day for a month. After completion of 2 months, patients were started on Dexoren –S eye drops (Chloramphenicol 0.5% plus Dexamethasone Phosphate Indoco Remedies, Mumbai 0.1%) 4 times a day for the first month and then reduced to 2 times a day. Patients were on a maintenance dose of Lotepred eye drops (loteprednol, Sun Pharma, India) twice a day for next 6 months and once a day thereafter. The patients were followed up at 1 and 15 days after surgery and at 1, 3, 6, and 12 months postoperatively. In the eye bank, donor ECD and viability were evaluated in vitro with an inverted light microscope (Eye Bank KeratoAnalyzer, EKA-10, Konan Medical, Japan). Postoperatively the endothelium was photographed and evaluated in vivo using a Topcon SP3000p noncontact autofocus specular microscope (Topcon Corp, Tokyo, Japan) at 3, 6, and 12 months. Images of the central corneal window were analyzed and manually corrected and three measurements of ECD were averaged.

## 3. Results

**Figure 3:**
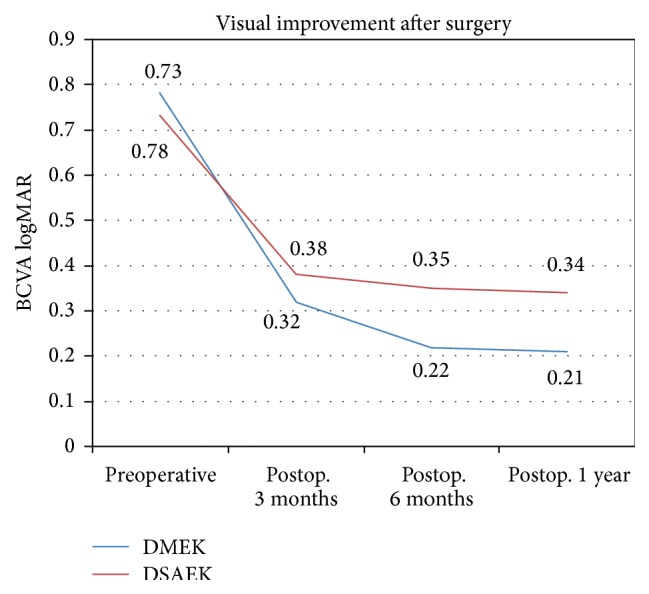
Visual improvement after DMEK and DSAEK.

**Figure 4:**
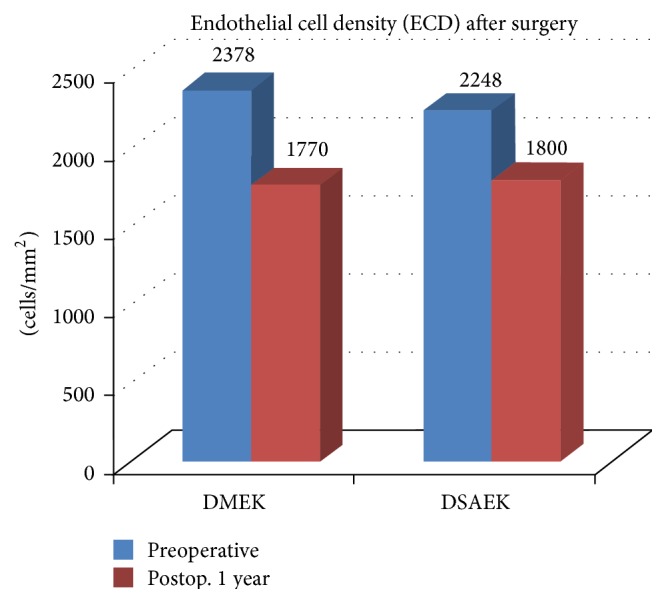
Endothelial cell density preoperative and postoperative in DMEK and DSAEK groups.

All patients (18 males, 12 females) with mean age of 55.12 ± 9.2 years (range 44–71 years) completed a 1-year follow-up following DMEK and DSAEK. Mean time interval between DSAEK and DMEK surgeries in fellow eyes was 12.1 ± 3.5 months (range 8–14 months). In our study, in eyes that underwent DMEK, the BCVA improved from preoperative values of 0.78 ± 0.35 logMAR to 0.32 ± 0.11 logMAR at 3 months and to 0.22 ± 0.1 logMAR (*P* < 0.001) at 6 months after surgery. In eyes that underwent DSAEK, the BCVA improved from preoperative values of 0.73 ± 0.31 logMAR to 0.38 ± 0.22 logMAR at 3 months and to 0.35 ± 0.12 logMAR (*P* < 0.001) at 6 months after surgery (Figure 3, Table 1). At one year after surgery, the BCVA was maintained at 0.21 ± 0.12 logMAR and 0.34 ± 0.1 logMAR, respectively, after DMEK and DSAEK. Eyes undergoing DMEK showed a statistically better improvement in visual acuity than the fellow eyes undergoing DSAEK (*P* < 0.05) (Table 2). 24 eyes (80%) achieved a BCVA of 0.18 logMAR or better 3 months after DMEK which was maintained at 1 year. In eyes that underwent DSAEK, 66.67% (20 eyes) achieved a BCVA of 0.18 logMAR or better at 3 months after surgery which was maintained at 1 year. There was no statistical significant difference in the preoperative BCVA (*P* = 0.2) in the two groups. Three eyes in the DMEK group and 3 eyes in the DSAEK group had preexisting cystoid macular edema (CME) which was contributory to the low postoperative visual acuity. Eyes with CME showed a modest improvement in visual acuity after surgery, from preoperative values of 0.02 ± 0.02 logMAR and 0.03 ± 0.12 logMAR, respectively, in the DSAEK and DMEK groups to postoperative values of 0.08 ± 0.12 logMAR and 0.09 ± 0.12 logMAR units. All eyes were pseudophakic with a posterior chamber intraocular lens implanted previously. ECD of donor corneas in DMEK group was 2378 ± 172 cells/mm^2^ and decreased to 1775 ± 121 cells/mm^2^ at 6 months after surgery (*P* < 0.001). ECD of donor corneas in DSAEK group was 2248 ± 142 cells/mm^2^ and decreased to 1806 ± 141 cells/mm^2^ at 6 months after surgery (*P* < 0.001) (Figure 4, Table 3). At one year after surgery the ECD was 1770 ± 124 cells/mm^2^ and 1800 ± 140 cells/mm^2^, respectively, after DMEK and DSAEK. A mean endothelial cell loss of 24% and 21% was observed 1 year after DMEK and DSAEK, respectively. There was no statistical significance difference (*P* = 0.08) in the endothelial cell loss observed between the two groups. The mean preoperative central corneal thickness (CCT) was 552.3 ± 34.1 *μ* and 561.24 ± 25.1 *μ* in the DMEK and DSAEK groups, respectively (*P* = 0.07). The mean thickness of the lenticule implanted in DMEK and DSAEK groups was, respectively, 11.18 ± 1.4 *μ* and 91.1 ± 10.1 *μ*. The mean postoperative central corneal graft thickness as measured by AS-OCT was 10.12 ± 1.2 *μ* and 81.11 ± 11.2 *μ* in the DMEK and DSAEK groups, respectively, at 6 months. The mean postoperative CCT observed in the DSAEK group of 621.19 ± 44.1 *μ* was significantly higher than in the DMEK group of 580.12 ± 14.1 *μ* (*P* < 0.001) Table 4.

After DMEK, 3 eyes (10%) showed partial dehiscence of the DM and required air injections in the early postoperative period. After DSAEK, none of the eyes required repeat air injections. In our study, there was loss of two donor corneas while stripping the DM in the DMEK group. No other complications like graft rejection were noted during the follow-up period.

## 4. Discussion

When DSEK was first introduced by F. W. Price Jr. and M. O. Price in 2005, it was observed that the technique maintained the structural integrity of the cornea and also provided rapid visual recovery for the patients [11]. Subsequently, in 2006, DMEK was introduced by Melles and associates [14] when it was concluded that the new surgical technique would provide quick visual rehabilitation to the patients without compromising on the endothelial cell survival. The usage of PK for patients with corneal endothelial disorders has rapidly declined since then. DSAEK is a surgical technique that is widely practised these days. DMEK is a surgical technique which is performed to a limited extent because of problems associated with the donor preparation and difficulties associated with unfolding of the EDM in the anterior chamber. These issues could be resolved by incorporating standardized techniques for donor preparation and insertion as described by Dapena et al. [27] and Kruse et al. [26]. Currently DSAEK remains the surgical procedure of choice for treating endothelial corneal disorders. It is a generally accepted fact that DSAEK allows for a good donor preparation and easier manipulation in the anterior chamber because of the higher stability of the comparatively thick graft. Further, the availability of the new graft insertion devices for DSAEK [29, 30] makes it a more sought-after procedure in comparison with DMEK. Nevertheless, the thickness of the posterior lamella in DSAEK, though decreasing with time [31] seems to have an influence on the visual outcome. The poorer visual acuity in DSAEK has been attributable to the presence of a stromal lamella which seems to cause posterior astigmatism, a hyperopic shift [31–34] or at least higher-order optical aberrations [35]. These findings have also been consistent with the observations in our study where the patients in the DSAEK group had a lesser postoperative visual acuity when compared to the patients in the DMEK group. In our study, also the postoperative central corneal thickness observed was higher in the DSAEK group. Also, a recently described data by Neff et al. [36] reported that grafts with a thickness of ≤131 *μ*m showed a statistically significant improvement in Best Spectacle Corrected Visual Acuity (BSCVA) compared to thicker grafts. Another data described by Busin, who introduced the ultra-thin DSAEK (UT-DSAEK) with a graft thickness of 73 ± 14 *μ*m, reported better functional outcome when compared to conventional DSAEK. Thus, DMEK, where the donor graft does not have a posterior lamella, is thought to provide a better functional outcome.

In this study, eyes with DMEK were found to have a better visual rehabilitation when compared to the fellow eyes which underwent DSAEK similar to that observed by Goldich et al. [37]. In our study, the visual outcome after DMEK in patients was similar to previously published data by Ham and associates [16] and Droutsas and associates [38], who reported a visual acuity of 20/40 or better in 95% and 96% of patients, respectively, and a visual acuity of 20/25 or better in 75% and 74% of patients, respectively, 6 months after surgery. Price and associates [39] observed a visual acuity of 20/40 or better in 94% of patients and a visual acuity of 20/25 or better in 63% of patients 3 months after surgery. Tourtas and associates [3] observed a visual acuity of 20/40 or better in 95% and 43% of patients, 6 months after DMEK and DSAEK, respectively. The percentage of visual acuity of 20/40 or better 6 months after DSAEK has been observed to vary between 80% and 97% [40]. Another factor of paramount importance which decides the success of a posterior lamellar keratoplasty is endothelial cell survival. In our study, the endothelial cell loss after DMEK and DSAEK was 24% and 21%, respectively, 1 year after surgery. Tourtas and associates [3] reported an endothelial cell loss of 41% in the DMEK group and 39% in the DSAEK group. The endothelial cell loss in our study was similar to the recently published data after DMEK by Melles group [16, 18, 37, 41], with a range of 19% to 33% 6 months after surgery and that by Goldich et al. [37]. In our study, the endothelial cell loss in the DMEK group may be attributable to increased endothelial cell loss caused during donor preparation and manipulation of the DM in the anterior chamber during the surgery. The endothelial cell loss in the DSAEK group was comparable to other studies [22, 42]. In our study, there was loss of two donor corneas while stripping the DM in the DMEK group. This result is similar to the observations in a study conducted by Tourtas and associates [3]. Our observation differs in this regard, from a data published by Price and associates [39] in which 12 of 72 eyes could not be stripped successfully. The lower incidence in our study may be attributable to the use of 2 forceps for the stripping procedure. During stripping, the EDM gets stretched and folded. Using 1 forceps, as observed by Price and associates [39], can easily cause the EDM to rupture. In contrast, using 2 forceps allows lifting of the EDM without folds and minimizes the traction forces [26]. In cases of DMEK, preparation of graft immediately prior to transplantation is possible with the help of commercially available, inexpensive devices whereas larger investments are required for donor preparation in such situations in DSAEK cases. A drawback of DMEK, based on the findings in this study, is the higher rebubbling rate after surgery (10%), as compared to DSAEK (0%). Despite the higher rebubbling rate, the functional outcome after DMEK remained unaffected. However, the increased rate of rebubbling is found to be associated with increased postoperative effort in terms of additional air injections in the early postoperative period. Based on the observations in our study, we feel that standardizing the DMEK surgical technique, would make the technique safe and patient-friendly. Also the adhesive property of the graft used in DMEK needs to be evaluated.

An advantage of DMEK is that the two procedures DMEK and DALK (Deep Anterior Lamellar Keratoplasty) can be combined, wherein a single cornea can be used twice. This procedure is also known as split cornea transplantation. This therefore reduces the need for corneal tissue and thereby and limits shortage of grafts. The cost and time involved in procuring the corneal tissue are also reduced. Data published by Heindl and associates [43, 44] suggest that this approach is feasible, reducing the need and the cost of corneal tissue by 45%. Our study, however, had its own limitations as being a retrospective study; neither the patients nor the professionals were blinded to the procedures and this needs to be considered. Also, in our study, the first eye underwent DSAEK and the later fellow eye underwent DMEK as we were refining our surgical technique of DMEK in the earlier phase while DSAEK was being performed. Nevertheless, compared to other studies [34], this study is the largest case series comparing DSAEK and DMEK in fellow eyes in the Indian population with a one-year follow-up.

## Figures and Tables

**Table 1:** Showing visual improvement after DMEK and DSAEK.

	Peroperative BCVA (logMAR)	6 months postop. BCVA (logMAR)	*t*-value	*P* value
DMEK	0.78 ± 0.35	0.22 ± 0.1	6.68	<0.001
DSAEK	0.73 ± 0.31	0.35 ± 0.12	6.12	<0.001

Paired *t*-test.

**Table 2:** Comparing visual improvement after DMEK and DSAEK.

BCVA (logMAR) 6 months after DMEK	BCVA (logMAR) 6 months after DSAEK	*Z*-score	*U*-value	*P* value
0.22 ± 0.1	0.35 ± 0.12	−5.7881	58	<0.05

Mann-Whitney *U* value test.

**Table 3:** Showing endothelial cell density (ECD) after DMEK and DSAEK.

	ECD preoperatively (cells/mm^2^)	ECD postop. 1 year (cells/mm^2^)	*t*-value	*P* value
DMEK (*n* = 30)	2378 ± 172	1770 ± 124	5.48	<0.001
DSAEK (*n* = 30)	2248 ± 142	1800 ± 140	5.89	<0.001

Paired *t*-test.

**Table 4:** Showing preoperative corneal and lenticule thickness and postoperative corneal and graft thickness in the DMEK and DSAEK groups.

Thickness in *µ*	DMEK group	DSAEK group	*P* value
Preoperative CCT	552.3 ± 34.1	561.24 ± 25.1	0.07
Preoperative lenticule thickness	11.18 ± 1.4	91.1 ± 10.1	
Postoperative CCT	580.12 ± 14.1	621.19 ± 44.1	<0.001
Postoperative CCGT	10.12 ± 1.2	81.11 ± 11.2	

CCT: central corneal thickness; CCGT: central corneal graft thickness.
